# Potential of *Tecoma stans* as an Insectary Plant for the Mirid Predator *Tytthus chinensis* (Hemiptera: Miridae): Effects on Longevity and Reproductive Development

**DOI:** 10.3390/insects17101021

**Published:** 2026-10-02

**Authors:** Wittaya Singsanan, Kazumu Kuramitsu, Apisit Thipaksorn, Panas Tumkiratiwong, Narisara Piyasaengthong

**Affiliations:** 1 Department of Zoology, Faculty of Science, Kasetsart University, Lat Yao, Chatuchak, Bangkok 10900, Thailand; 2 Faculty of Life and Environmental Sciences, University of Tsukuba, Tsukuba 305-8572, Ibaraki, Japan

**Keywords:** mirid predator, floral nectar, natural enemies, insectary plant, biological control, rice agroecosystem

## Abstract

The brown planthopper (*Nilaparvata lugens*, BPH) is a major pest in Asian rice ecosystems, and biological control by natural enemies is a key component of integrated pest management. Zoophytophagous mirid bugs, including *Tytthus chinensis* (Hemiptera: Miridae), play an important role as predators of BPH eggs and nymphs in Thailand. However, little is known about the potential of insectary plants to support the performance of *T. chinensis*. In this study, we evaluated the effects of *Tecoma stans* nectar on the longevity and reproductive development of *T. chinensis* under laboratory conditions. Adult longevity was compared among four feeding treatments: dry cotton, wet cotton, BPH eggs, and *T. stans* flowers. In addition, ovariole development was assessed under five feeding regimes combining BPH eggs and/or *T. stans* flowers. Access to *T. stans* nectar significantly extended adult longevity compared with the no-food treatments, and no significant difference in longevity was detected between predators provided with *T. stans* nectar and those provided with BPH eggs. Females provided with BPH eggs showed clear ovariole development, whereas those provided with *T. stans* nectar alone showed limited ovariole development. These results suggest that *T. stans* nectar may serve as an alternative food resource that supports adult survival, while access to prey appears to play an important role in reproductive development under the conditions tested. These laboratory findings support further evaluation of *T. stans* as a potential insectary plant for rice agroecosystems.

## Simple Summary

The mirid predator *Tytthus chinensis* is a natural enemy of the brown planthopper, an important rice pest in Asia. Providing alternative food resources may help this predator survive when prey is scarce. We investigated whether flowers of *Tecoma stans*, which provide nectar, could support adult survival and affect female reproductive development of *T. chinensis* under laboratory conditions. Access to *T. stans* flowers significantly extended adult longevity compared with the no-food treatments, and no significant difference in longevity was detected between predators provided with *T. stans* flowers and those provided with brown planthopper eggs. However, females provided with *T. stans* flowers alone showed limited ovariole development, whereas females provided with brown planthopper eggs showed clear ovariole development. These findings suggest that *T. stans* flowers may serve as an alternative food resource that supports adult survival, while access to prey appears to play an important role in ovariole development. Further field studies are needed to evaluate the potential of *T. stans* as an insectary plant in rice agroecosystems.

## 1. Introduction

Brown planthopper (BPH; *Nilaparvata lugens* (Stål), Hemiptera: Delphacidae) is a major pest across Asia, including Thailand. Both nymphs and adults feed on rice stems, causing plants to dry out and die over large areas, a phenomenon known as “hopper burn,” which ultimately leads to severe yield losses [1]. In addition, BPH transmits rice ragged stunt virus (RRSV) and rice grassy stunt virus (RGSV), both of which cause serious diseases in rice plants [2,3,4]. This pest can migrate long distances with the wind, allowing it to disperse widely across rice-growing regions [5,6]. Chemical control remains the primary strategy for managing BPH. However, reliance on insecticides has several drawbacks. Continuous application can lead to the rapid development of resistance in BPH populations [7], and insecticides may also cause negative side effects on natural enemies, resulting in pest resurgence and recolonization [8]. Some insecticides have even been reported to enhance BPH survival and reproduction [9]. Moreover, migration of resistant BPH populations can further diminish the effectiveness of chemical control [10]. The use of resistant rice cultivars is an alternative management approach; however, its effectiveness can be compromised [11], as BPH populations can adapt to resistant cultivars [12].

In this context, biological control using natural enemies remains a critical component of integrated pest management (IPM) for BPH in rice-field ecosystems. In Asian rice systems, zoophytophagous mirid bugs (Hemiptera: Miridae) are important natural enemies of BPH, preying on eggs and nymphs and contributing to the suppression of BPH populations [13,14]. In Thailand, the green mirid bug *Cyrtorhinus lividipennis* Reuter, a well-known predator of BPH, and another mirid species, *Tytthus chinensis* (Stål), are important natural enemies of BPH [15,16,17].

A study by Ritmontree et al. [18] demonstrated that *T. chinensis* is effective in controlling BPH populations. In addition, *T. chinensis* can prey on up to 20 eggs per day of *Sogatella furcifera* (Hemiptera: Delphacidae), a planthopper pest species closely related to BPH [19]. According to Bai et al. [20], *T. chinensis* and *C. lividipennis* exhibit different levels of tolerance to heat stress, with *T. chinensis* showing superior thermal tolerance. Considering that global warming may accelerate BPH outbreaks [21], *T. chinensis* and *C. lividipennis* may serve as complementary or alternative biological control agents under future climate scenarios. Therefore, it is important to study *T. chinensis* alongside the well-studied *C. lividipennis*.

Within the framework of IPM, insectary plants can contribute to sustainable pest management by strengthening the functional role of natural enemies and reducing reliance on chemical pesticides. The availability of food resources in the field strongly influences the performance of natural enemies. Therefore, enhancing the abundance and performance of natural enemies can be achieved through habitat management, such as the incorporation of insectary plants [22,23,24,25,26,27]. These flowering plants provide essential resources, including nectar, pollen, and shelter, which enhance the attraction, survival, development, and retention of beneficial natural enemies, including nectar-feeding parasitoids and zoophytophagous mirid bugs [28].

To identify suitable insectary plants, it is essential to first evaluate their effects on target natural enemies through controlled feeding experiments. To date, research on the effects of insectary plants on mirid performance has been conducted primarily on *C. lividipennis*. For example, Zhu et al. [29] assessed how several flowering plant species influence the survival and predation efficiency of *C. lividipennis*. In contrast, despite its importance as a predator of BPH, limited information is available on the effects of insectary plants or floral resources on the longevity and reproductive development of *T. chinensis.* Given that floral nectar has been shown to enhance the survival of *C. lividipennis* [29], it is reasonable to hypothesize that *T. chinensis*, which is closely related to *C. lividipennis* and shares similar ecological traits, may also benefit nutritionally from nectar. Therefore, in this study, we evaluated the effects of *Tecoma stans* (L.) Juss. ex Kunth (Lamiales: Bignoniaceae) nectar on *T. chinensis*, focusing specifically on adult longevity and reproductive development.

Yellow elder, *T. stans*, is a perennial shrub native to tropical and subtropical America but has become naturalized across Africa, the Pacific, and Asia, including Thailand [30,31]. The plant has received considerable attention for its medicinal and antibacterial properties [32,33,34,35]. *T. stans* provides nectar that attracts bees and other flower-visiting insects [36,37,38].

Two characteristics make *T. stans* a plausible candidate insectary plant for Thai rice landscapes. First, it flowers for extended periods, tolerates drought and high temperatures, and can grow under a wide range of soil conditions [38]. Consequently, *T. stans* nectar may be available during periods when planthopper prey are scarce. Second, *T. stans* is already widely established and grown as an ornamental plant in Thailand [39], although it is considered an invasive alien plant in some other parts of its introduced range.

Accordingly, this study evaluated the potential of *T. stans* as a food resource for *T. chinensis* under laboratory conditions. Specifically, we compared adult longevity among four feeding treatments (dry cotton, wet cotton, BPH eggs, and *T. stans* flowers) and assessed ovariole development under five feeding regimes involving BPH eggs and *T. stans* flowers. These experiments aimed to determine whether access to *T. stans* flowers supports adult survival and how different feeding regimes affect ovariole development, providing a basis for further evaluation of *T. stans* as a potential insectary plant.

## 2. Materials and Methods

### 2.1. Insect Rearing and Plant Cultivation

The original colony of the mirid predator *Tytthus chinensis* and the brown planthopper (BPH), *Nilaparvata lugens*, were obtained from the Rice Department, Bangkok, Thailand (Supplementary Figure S1). The mirids were reared in a cage (40.5 × 40.5 × 60 cm) containing 3–5-day-old rice seedlings (*Oryza sativa* f. *indica* ‘Pathum Thani 1′) with BPH eggs on the leaf sheath. The rice variety ‘Pathum Thani 1 (PT1)’ is recommended for cultivation in irrigated zones of the central region of Thailand [40]. Mass rearing was conducted in an open rooftop environment under natural temperature and light conditions at the Department of Zoology (13°50′46.1″ N, 100°34′16.9″ E), Faculty of Science, Kasetsart University, Thailand. Plants of yellow elder, *Tecoma stans*, were obtained from Nonthaburi Province, Thailand, and cultivated in 45 cm diameter pots under the same environmental conditions as the insect colonies. To prevent potential pesticide contamination affecting the test insects, flowering branches were removed, and plants were regrown for six months until flowering before the experiment.

### 2.2. Laboratory Conditions and Experimental Preparation

All experiments were conducted under controlled laboratory conditions (26 ± 1 °C, 60 ± 10% RH, 12 L:12 D photoperiod, a light intensity of approximately 2.5 klx, with the light phase from 06:00 to 18:00 h). BPH-infested rice seedlings were prepared by releasing approximately 500 adult BPH into trays (16 × 30 cm) containing approximately 300 rice seedlings aged 3–4 days for 2–3 nights. The seedlings were approximately one week old when used in the experiments. The exact number and age of individual BPH eggs were not quantified. The rice seedlings infested with BPH eggs were used for the experiments (hereafter BPH-infested rice seedlings). Prior to the experiments, the roots of each infested rice seedling were washed and wrapped with moist cotton during the trials. *Tecoma stans* flowers were prepared by cutting peduncles, each bearing three open flowers, and the cut surface of the peduncle was wrapped with moist cotton before use. The moist cotton was used to maintain the hydration of the plant material and remained accessible to *T. chinensis* during the experiments.

### 2.3. Longevity of the Mirid Predator Tytthus chinensis

Late-instar nymphs (fourth and fifth instars) were randomly collected from the rearing colony and maintained in holding containers until adult emergence. Before adult emergence, the rice seedlings in the holding containers were replaced with seedlings free of BPH eggs to prevent access to BPH eggs prior to treatment assignment. Newly emerged adults of each sex, within 6 h after emergence, were randomly allocated among the four feeding treatments, forming eight experimental groups. Each adult was individually introduced into a transparent plastic rearing container consisting of a cylindrical cup covered with a plastic dome (9.5 cm in diameter and 19.5 cm in height). Each container housed a single *T. chinensis* together with the feeding materials. The experimental containers were placed within the designated laboratory area, and their positions were not randomized or rearranged during the experiment.

The longevity experiments were conducted under four feeding conditions: (1) dry cotton (mean weight 0.62 g), (2) wet cotton moistened with 3 mL of water, (3) ten rice seedlings infested with BPH eggs, and (4) three intact open flowers of *T. stans*. Nectar was potentially available from both a nectary disc at the base of the ovary and extrafloral nectaries on the calyx [38,41]. These nectar sources are hereafter collectively referred to as *T. stans* nectar. The amount of nectar available to individual predators was not quantified.

The infested rice seedlings were replaced every 5–7 days. The removed seedlings were retained and monitored for subsequent BPH nymphal emergence to confirm that viable eggs were still present at the time of replacement. The *T. stans* flowers were replaced every 3 days to maintain resource quality. Each treatment consisted of 25–27 replicates for each sex. Insect survival was monitored twice daily at 08:00 and 16:00 h until all individuals had died (maximum observation period: 46 days).

### 2.4. Reproductive Development of the Mirid Predator Tytthus chinensis

The reproductive development of *T. chinensis* females fed on BPH eggs and/or *T. stans* nectar was examined by dissection. As in the longevity experiment, the rice seedlings in the holding containers were replaced with seedlings free of BPH eggs before adult emergence. Newly emerged females within 6 h after emergence were used in the reproductive development experiment. Females were individually reared using the same methods and under the same conditions described above and assigned to five feeding treatments: (1) BPH-infested rice seedlings for 3 days, (2) BPH-infested rice seedlings for 6 days, (3) BPH-infested rice seedlings for 3 days followed by *T. stans* flowers for 3 days, (4) *T. stans* flowers for 3 days followed by BPH-infested rice seedlings for 3 days, and (5) *T. stans* flowers for 6 days. In certain feeding experiments, the design consisted of two feeding phases, each lasting 3 days. Females were exposed to feeding regimes lasting either 3 or 6 days. In the sequential treatments, the 6-day period was divided into two equal 3-day phases to test whether the order of exposure to BPH eggs and *T. stans* flowers affected ovariole development. A treatment in which females received BPH eggs for 3 days was included for comparison with females fed BPH eggs for 6 days, allowing temporal changes in ovariole development to be assessed.

At the end of the feeding period, females were collected and preserved in 1.5 mL microcentrifuge tubes containing 95% ethanol. The number of ovarioles in each female was counted by dissection, and the straight-line length of each ovariole was measured using a Nikon SMZ800N stereomicroscope equipped with a digital imaging system and NIS-Elements D4.13.00 software. The mean ovariole length for each female was then calculated from all measured ovarioles. Females without developed ovarioles were recorded as zero for ovariole number, whereas no ovariole-length value was assigned to individuals without a measurable ovariole. Each treatment consisted of 10–11 females.

### 2.5. Statistical Analysis

Normality and homogeneity of variance were assessed using the Shapiro–Wilk and Levene’s tests, respectively. Because the assumptions for parametric analysis were not met for adult longevity, the effects of feeding treatment, sex, and their interaction were analyzed using the Scheirer–Ray–Hare test, a nonparametric extension of the Kruskal–Wallis test for factorial designs [42]. Ovariole number was analyzed using the Kruskal–Wallis test because the assumptions for parametric analysis were also not met. Where pairwise comparisons were required, Dunn’s multiple-comparison test with Bonferroni correction was used.

Mean ovariole length among the four BPH-containing feeding regimes was analyzed using one-way ANOVA. For this analysis, normality of the model residuals was assessed using the Shapiro–Wilk test and a normal Q–Q plot, and homogeneity of variance was assessed using Levene’s test. These diagnostic assessments suggested that the assumptions for ANOVA were reasonably met. The *T. stans* nectar-only treatment was excluded from this analysis because only one female had a measurable ovariole. All statistical analyses were performed in R (version 4.4.2; R Foundation for Statistical Computing, Vienna, Austria) [43] using the packages *rcompanion*, *FSA*, and *car*. A *p* value < 0.05 was considered statistically significant.

## 3. Results

### 3.1. Effect of Different Food Sources on the Longevity of the Mirid Predator Tytthus chinensis

**Figure 1:**
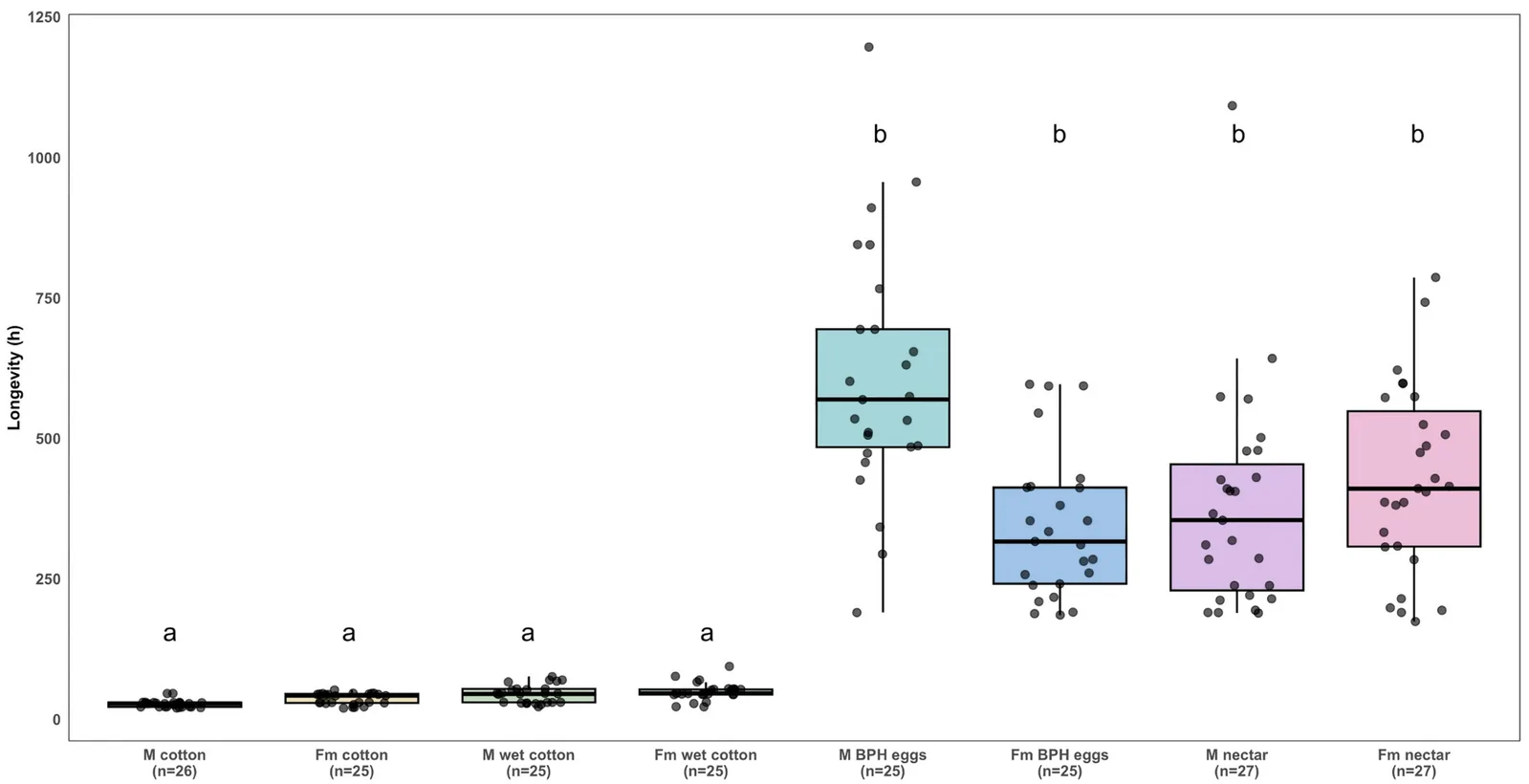
Longevity (in hours) of male (M) and female (F) *Tytthus chinensis* under different feeding treatments. Box plots represent the median (horizontal line), interquartile range (box), and individual data points (dots). Treatments included dry cotton, wet cotton, BPH eggs, and *Tecoma stans* nectar. Different letters indicate significant differences among the eight combinations of feeding treatment and sex based on Dunn’s multiple-comparison test with Bonferroni correction (*p* < 0.05).

The longevity of adult *T. chinensis* varied depending on the feeding treatment provided (Figure 1). The Scheirer–Ray–Hare test indicated that feeding treatment had a significant effect on longevity (Df = 3, Sum Sq = 563,113, H = 160.17, *p* < 0.0001), whereas sex had no significant effect (Df = 1, Sum Sq = 1, H = 0.00, *p* = 0.984), and the interaction between feeding treatment and sex was not significant (Df = 3, Sum Sq = 24,392, H = 6.938, *p* = 0.074). For the pairwise comparisons shown in Figure 1, the eight combinations of feeding treatment and sex were treated as eight groups and compared using Dunn’s multiple-comparison test with Bonferroni correction. In the unfed treatments, individuals provided with dry cotton showed the shortest median longevity: 28 h (IQR: 22.00–29.75 h) for males and 42 h (IQR: 29.00–45.00 h) for females. In the wet-cotton treatment, maximum longevity reached 76 h in males and 94 h in females; however, longevity did not differ significantly between the dry- and wet-cotton groups (*p* > 0.05).

**Figure 2:**
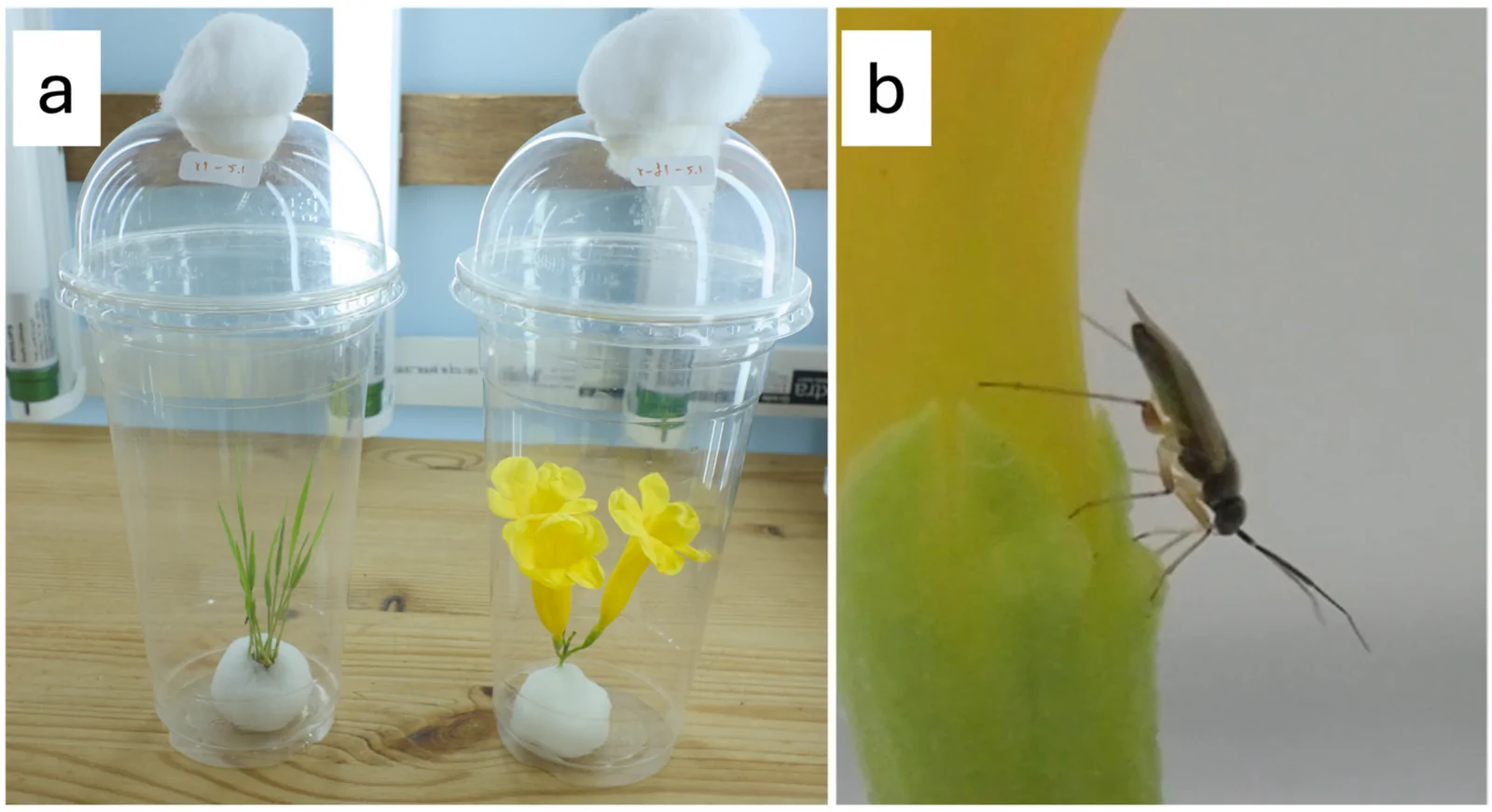
Experimental setup and nectar feeding by adult *Tytthus chinensis*. (**a**) Representative experimental containers showing the *Tecoma stans* flower treatment and the BPH-infested rice seedling treatment. (**b**) Adult *T. chinensis* feeding on nectar at a visible nectary near the base of a *T. stans* flower during the feeding experiment. The image in (**b**) was extracted from Supplementary Video S1, which shows the same individual feeding on *T. stans* nectar.

Access to BPH eggs or *T. stans* nectar substantially increased longevity relative to the unfed treatments. Insects provided with BPH eggs showed prolonged survival, with males showing a higher median longevity (median: 569.00 h; IQR: 484.00–694.00 h) than females (median: 316.00 h; IQR: 241.00–412.00 h), although the difference was not statistically significant (Dunn’s multiple comparison test, *p* > 0.05). Adults supplied with *T. stans* nectar also exhibited extended longevity. Predators were directly observed feeding on nectar droplets from the extrafloral nectaries on the calyx of *T. stans* (Figure 2). Females showed a higher median longevity (median: 410.00 h; IQR: 307.00–548.00 h) than males (median: 354.00 h; IQR: 229.00–453.50 h), but the difference was not statistically significant (Dunn’s multiple comparison test, *p* > 0.05). No significant difference in longevity was detected between groups provided with BPH eggs and those provided with *T. stans* nectar for either sex (Dunn’s multiple-comparison test, *p* > 0.05).

### 3.2. Effect of Different Food Sources on the Reproductive Development of Female Tytthus chinensis

**Figure 3:**
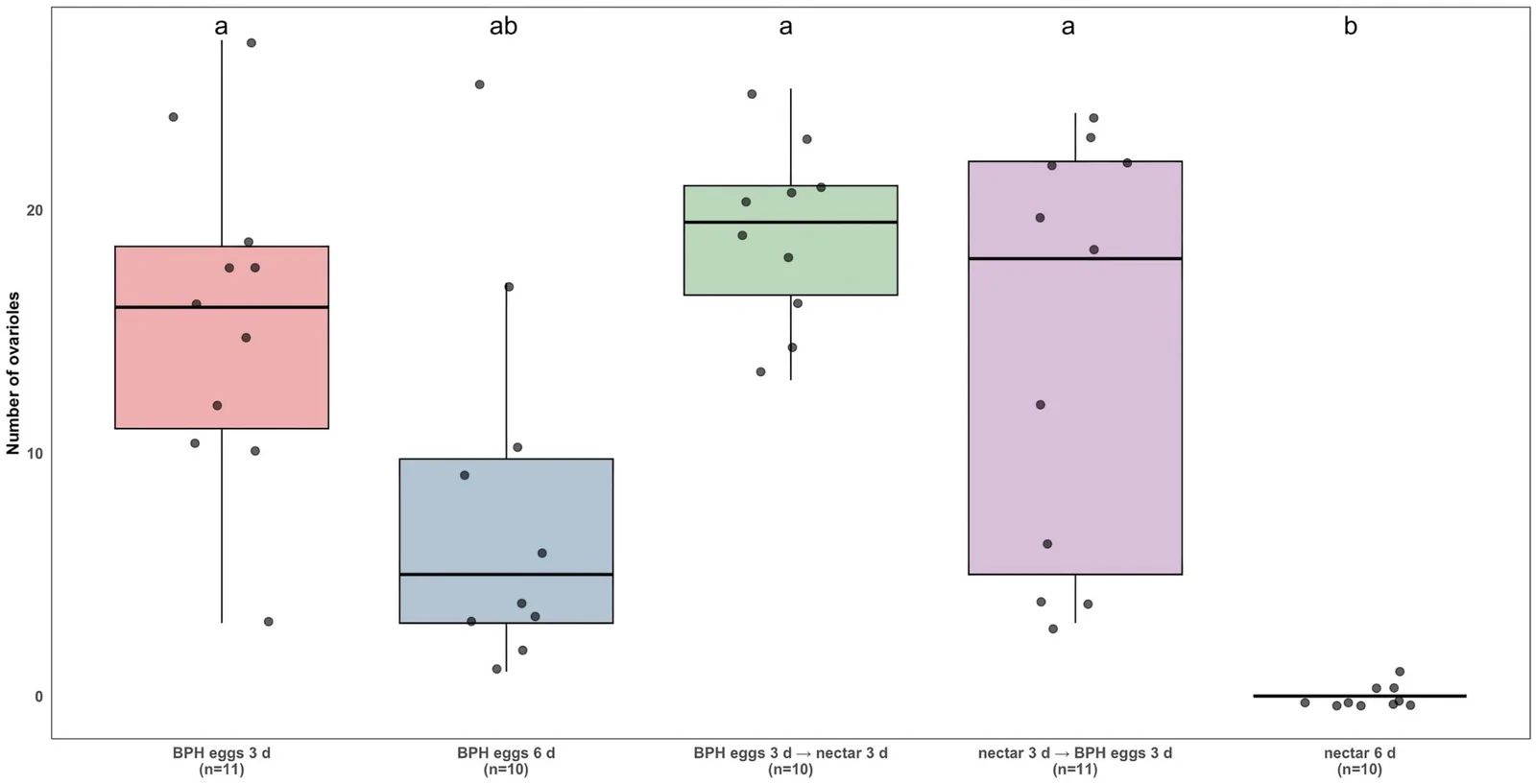
Number of ovarioles per female *Tytthus chinensis* under five different feeding treatments. Each dot represents the total number of ovarioles counted in a single individual. Boxes show the interquartile range (IQR), the horizontal line indicates the median, and whiskers represent the minimum and maximum values. Different letters (a, b) denote statistically significant differences among treatments based on Dunn’s post hoc test (*p* < 0.05).

Ovarioles were observed in all individuals that were fed BPH eggs during the experimental period (treatments (1)–(4); Figure 3, Supplementary Figure S2). The number of ovarioles ranged from single digits to more than 20, depending on the feeding treatment (Figure 3). Among the egg-fed groups, individuals in treatments (1), (3), and (4) showed similar ovariole numbers, with median (IQR) values of 16.00 (11.00–18.50), 19.50 (16.50–21.00), and 18.00 (5.00–22.00), respectively, and maximum ovariole counts of 27, 25, and 24.

Statistical analysis using the Kruskal–Wallis test followed by Dunn’s multiple comparison test indicated that the *T. stans* nectar-only treatment (treatment 5) differed significantly from treatments 1, 3, and 4 (χ^2^ = 30.1, df = 4, *p* < 0.001). However, the nectar-only group (treatment 5) did not differ significantly from treatment (2), in which females were fed BPH eggs for six days (*p* > 0.05). In treatment (2), females fed BPH eggs for six consecutive days had a median ovariole count of 5 (IQR: 3.00–9.75), with a minimum of 1 and a maximum of 25. Although the maximum value was comparable to those in the other egg-fed treatments, the median value was markedly lower. In the *T. stans* nectar-only treatment (treatment 5), ovariole development occurred in only one of the ten individuals, and that female produced a single ovariole.

**Figure 4:**
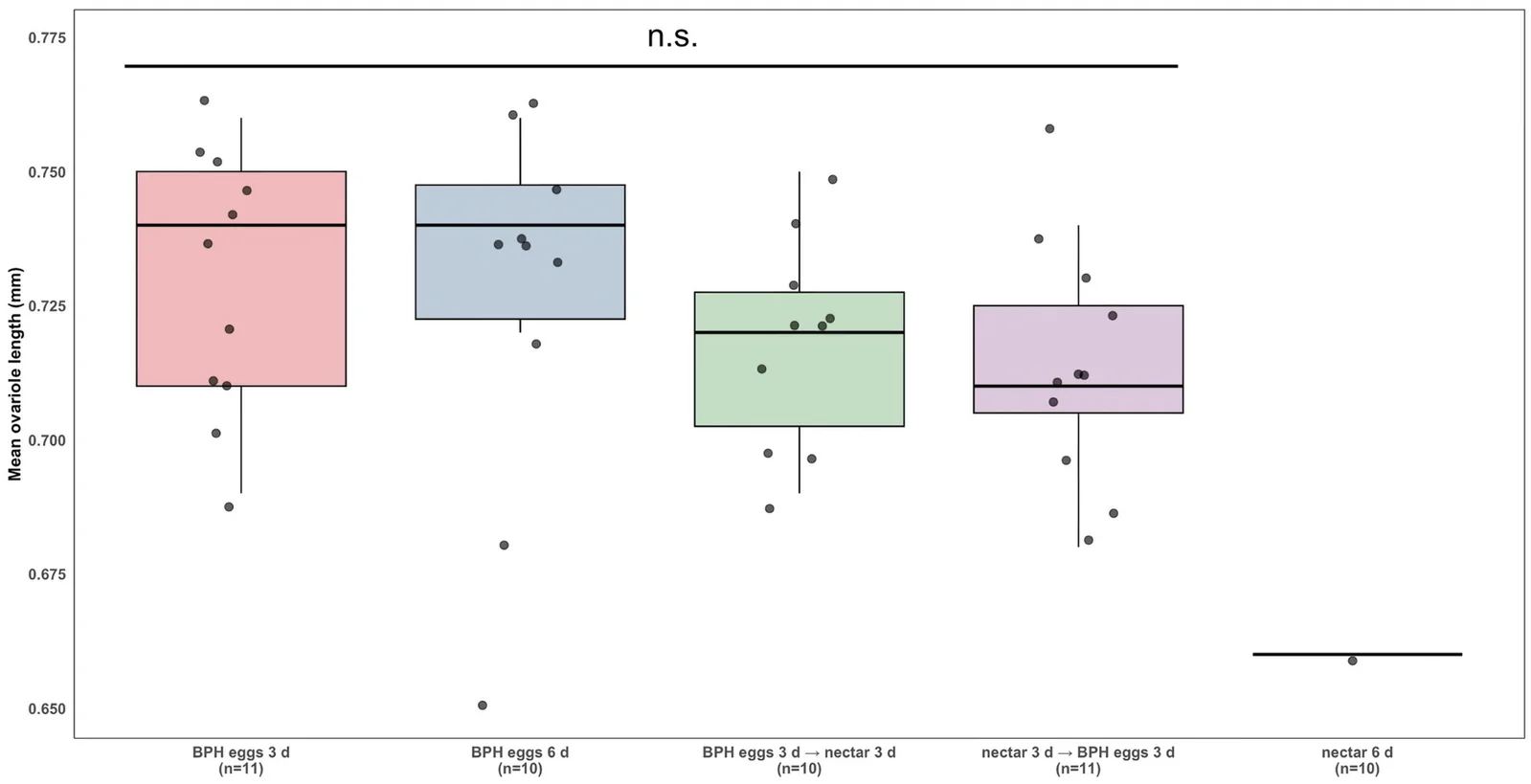
Mean ovariole length (mm) of female *Tytthus chinensis* under five dietary treatments. Each dot represents the mean ovariole length of an individual. Boxes indicate the interquartile range (IQR), the horizontal line indicates the median, and whiskers represent the minimum and maximum values. One-way ANOVA revealed no significant differences (n.s.) in mean ovariole length among treatments 1–4 (F(3, 38) = 0.787, *p* = 0.509). Only one female in the *T. stans* nectar treatment had a measurable ovariole. Therefore, this treatment was excluded from the statistical comparison of mean ovariole length, although the value for this individual is shown descriptively.

Ovariole length measurements showed that females in the egg-fed treatments (treatments 1–4) had average ovariole lengths ranging from 0.65 to 0.76 mm, with median values between 0.71 and 0.74 mm (Figure 4, Supplementary Figure S2). One-way ANOVA revealed no significant difference in mean ovariole length among the four BPH-containing treatments (F(3, 38) = 0.787, *p* = 0.509). In the *T. stans* nectar-only treatment (treatment 5), only one female developed a single ovariole with a length of 0.66 mm; therefore, no statistical comparison was possible.

## 4. Discussion

### 4.1. The Potential of Tecoma stans as a Food Source for Tytthus chinensis

This study demonstrates that *Tecoma stans* nectar can serve as an alternative food resource for adult *Tytthus chinensis*, as access to nectar significantly increased longevity relative to the no-food treatments (dry cotton and wet cotton) in both males and females. No significant difference in longevity was detected between predators provided with *T. stans* nectar and those provided with BPH eggs. Although males provided with *T. stans* nectar had a lower median longevity than those provided with BPH eggs, this difference was not statistically significant. Individuals provided with *T. stans* nectar nevertheless had significantly greater longevity than those in the dry- and wet-cotton treatments.

Plant-derived food resources, particularly sugars, can enhance survival in predatory insects [44]. For example, access to flowering plants significantly increased the longevity of the predatory mirid *Cyrtorhinus lividipennis* compared with water or no-food controls [29]. *T. stans* nectar has been reported to contain predominantly glucose (57.5%) and fructose (41.3%), with a small proportion of sucrose (1.2%) [45]. This reported sugar composition may partly explain the extended longevity observed in the present study; however, the sugar composition of the nectar used in our experiment was not measured.

Another possible explanation lies in the floral structure. In the flowers used in this study, nectar was potentially available from two distinct sources: a nectary disc located at the base of the ovary, within the corolla tube [41], and extrafloral nectaries on the calyx [38], where nectar droplets were visible throughout the experiment. The availability of nectar from more than one site may therefore have contributed to the extended longevity recorded under the *T. stans* nectar treatment.

In contrast, the longevity of predators supplied only with moist cotton as a water source was not significantly greater than that of individuals provided with dry cotton. Although the moist cotton used to maintain the hydration of the *T. stans* flower peduncles was accessible to the predators, direct water intake from the cotton was not assessed and therefore cannot be excluded. However, the absence of a significant difference in longevity between the wet-cotton and dry-cotton treatments suggests that access to moisture alone was unlikely to account for the increased longevity observed in the *T. stans* nectar treatment. Humidity and water availability can influence insect survival [46,47,48]. Taken together, these results suggest that the longevity benefit associated with *T. stans* nectar is unlikely to be explained by moisture availability alone. 

Our results indicate that *T. chinensis* follows a pattern previously documented in related mirids. However, the longevity observed in the present study was considerably longer than that reported for *Cyrtorhinus lividipennis*. Whereas access to flowers of *Tagetes erecta*, *Tridax procumbens*, *Emilia sonchifolia*, and *Sesamum indicum* extended longevity in *C. lividipennis* to 52.3–60.5 h [29], *T. chinensis* provided with *T. stans* nectar had an interquartile longevity range of 307–548 h in females and 229–454 h in males. Because these values were obtained under different experimental conditions, this comparison should be interpreted descriptively. The prolonged survival supported by *T. stans* nectar may be particularly relevant during short periods when BPH eggs are scarce. Wada et al. [49] reported a pre-oviposition period of approximately 4.1 d in tropical BPH populations under 25 ± 1 °C. Such a delay could potentially create a brief period following BPH colonization during which egg prey are scarce, and alternative food resources such as *T. stans* nectar may help sustain adult *T. chinensis* until BPH eggs become available. However, whether such a temporal gap occurs under field conditions and coincides with the arrival of *T. chinensis* remains to be tested. Furthermore, the present study did not include a rice seedling-only treatment or a combined BPH egg and *T. stans* flower treatment. Therefore, the effects of rice seedlings alone and the combined effects of the two food resources remain to be evaluated.

Overall, these findings indicate that *T. stans* nectar can support the adult survival of *T. chinensis* under laboratory conditions and suggest its potential as an alternative food resource during periods of prey scarcity. Its potential value as an insectary plant for supporting predator persistence and biological control in rice agroecosystems requires further evaluation under field conditions.

### 4.2. Comparative Effects of BPH Egg Predation and Tecoma stans Nectar Feeding on the Reproductive Development of Tytthus chinensis

The present results demonstrate differential effects of prey and non-prey food resources on the reproductive development of the mirid predator *T. chinensis*. The findings indicate that BPH egg ingestion plays an important role in ovariole development in female *T. chinensis* under the conditions tested. All females that fed on BPH eggs, either continuously or in sequence with *T. stans* nectar, showed observable ovariole development. In contrast, females fed exclusively on *T. stans* nectar showed limited ovariole development, with only one individual having a single ovariole. The group fed on BPH eggs for 6 d had a lower median ovariole number (5.00, IQR: 3.00–9.75) than the group fed on BPH eggs for 3 d (16.00, IQR: 11.00–18.50). The reason for this pattern remains unclear and cannot be determined from the present data.

The limited ovariole development observed in the *T. stans* nectar treatment suggests that, although *T. stans* nectar can support adult survival, nectar alone may be insufficient to support ovariole development under the conditions tested. Differences in nutritional composition between the two food sources may have contributed to this pattern, although protein and other nitrogenous nutrients were not measured in either *T. stans* nectar or BPH eggs. Protein is nevertheless one biologically plausible factor because of its established role in insect reproductive development [50] and affects mating behavior and reproductive performance [51]. Protein functions both as an energy source and as a signaling component that regulates hormone biosynthesis and secretion, including the initiation of egg production through the amino acid–Target of Rapamycin (AA/TOR) and insulin signaling pathways [52]. Furthermore, protein availability has also been shown to influence sex pheromone production [53]. Together, these findings highlight the importance of adequate protein intake for normal reproductive function [54], even though predatory Hemiptera exhibit considerable dietary flexibility [55].

This finding further supports the importance of food quality in sustaining reproductive development in insects [56,57,58]. In contrast, inadequate nutrition may lead to energetic trade-offs in resource allocation, thereby compromising other essential physiological functions [59]. The limited ovariole development observed with *T. stans* nectar alone further suggests an important role of access to prey in ovariole development. Therefore, *T. chinensis* can be considered a zoophytophagous predator [60], highlighting its potential role in biological control in agricultural ecosystems.

Although this outcome might appear intuitive, it could not be assumed a priori. Within the Miridae alone, the capacity of plant-derived resources to sustain reproduction varies considerably among species, with *Deraeocoris signatus* neither surviving nor reproducing on cotton tips and cotton squares without prey [61], whereas *Nesidiocoris tenuis* completes development and reproduces on the plant tissues of *Cleome hassleriana* or *Sesamum indicum* in the complete absence of prey [62]. Whether *T. stans* nectar supports survival alone or can also contribute to reproductive development has important implications for its potential use in biological control. Because oviposition was not systematically assessed in this study, the effects of the different feeding regimes on fecundity and reproductive success remain to be determined. Based on our findings, *T. stans* may have potential to support the survival of *T. chinensis* during periods of prey scarcity, although its effect on predator persistence under field conditions remains to be tested.

### 4.3. Potential Role of Tecoma stans in the Indirect Outcomes of Increasing Habitat Diversity for Pest Control

Habitat diversification using insectary plants can provide food resources and refuges for natural enemies and thereby support biological control in agricultural landscapes [63]. In this context, *T. stans* may represent a potential insectary plant because of its prolonged flowering and availability of nectar resources [38]. Flowering plants can function as both resource and refuge providers for natural enemies. By increasing plant species richness and structural complexity, such habitat enhancements support more diverse natural enemy communities. Floral resources can attract and sustain a wide range of predators and parasitoids, thereby supporting biological control services in a more stable and sustainable manner [23,24,25,64]. However, the provision of alternative food does not necessarily translate directly into improved pest suppression, as its effects may depend on predator population responses, timing of food availability, and the quality of the alternative food [65].

Nevertheless, field-scale studies are required to determine whether *T. stans* can promote natural enemy diversity and pest suppression under real agricultural conditions. Such studies will be crucial for designing habitat management strategies that align with agroecological principles while ensuring practical benefits for farmers.

Overall, the present study demonstrates that *T. stans* nectar can serve as an alternative food source that enhances the survival of the mirid predator *T. chinensis*, although prey consumption appears to play an important role in reproductive development under the conditions tested. These findings suggest that *T. stans* nectar may support the survival of *T. chinensis* during periods of prey scarcity. Accordingly, *T. stans* may warrant further evaluation as an insectary plant for habitat management aimed at supporting biological control in rice agroecosystems.

## 5. Conclusions

This study demonstrates that access to *Tecoma stans* flowers can substantially extend the adult longevity of the mirid predator *Tytthus chinensis* under laboratory conditions. No significant difference in longevity was detected between predators provided with *T. stans* nectar and those provided with brown planthopper eggs. However, *T. stans* nectar alone resulted in limited ovariole development, whereas females provided with prey showed clear ovariole development. These findings suggest that *T. stans* nectar may support adult survival, while access to prey appears to play an important role in reproductive development under the conditions tested. Thus, the potential value of *T. stans* as an insectary plant may lie in supporting predator survival during periods of prey scarcity. Given its prolonged flowering period and widespread occurrence in Thailand, *T. stans* may therefore warrant further evaluation as a potential insectary plant for habitat management aimed at supporting biological control in rice agroecosystems. Further field studies are needed to determine whether the benefits observed under laboratory conditions translate into increased predator persistence or pest suppression under field conditions.

## Data Availability

The data presented in this study are available from the corresponding author upon reasonable request.

## Supplementary Materials

The following supporting information can be downloaded at: https://www.mdpi.com/article/10.3390/insects17101021/s1, Figure S1: Photographic documentation of the *Tytthus chinensis* rearing colony. (A) Rearing cage used to maintain the *T. chinensis* colony. (B–E) Representative adult *T. chinensis* individuals from the colony.; Figure S2: Representative photographs illustrating the dissection and observation of ovarioles in female *Tytthus chinensis*. (a) Abdomen of an adult female before dissection. (b) All ovarioles dissected from a single female, illustrating the material used for ovariole counting. (c–g) Representative ovarioles used for length measurements from females subjected to the five feeding treatments: (c) BPH eggs for 3 d, (d) BPH eggs for 6 d, (e) BPH eggs for 3 d followed by *T. stans* nectar for 3 d, (f) *T. stans* nectar for 3 d followed by BPH eggs for 3 d, and (g) *T. stans* nectar for 6 d.; Video S1: Adult *Tytthus chinensis* feeding on nectar at a visible nectary near the base of a *Tecoma stans* flower during the feeding experiment. The proboscis is clearly extended during feeding. A still image extracted from this video is presented in Figure 2b.
